# Hand-in-Hand–improving occupational health and quality of care in early education and care: study protocol for a cluster-randomized controlled trial

**DOI:** 10.3389/fpubh.2026.1981528

**Published:** 2026-10-05

**Authors:** Emily Morud, Markus Koch, Fiona Aanesen, Skender Elez Redzovic, Emil Sundstrup, Pieter Coenen, Ingrid Christensen, Ramus Kleppe, Suzanne L. Merkus

**Affiliations:** 1 Department of Work Psychology and Physiology, National Institute of Occupational Health, Oslo, Norway; 2 Centre for treatment of Rheumatic and Musculoskeletal Diseases (REMEDY), Diakonhjemmet Hospital, Oslo, Norway; 3 Department of Neuromedicine and Movement Science, Faculty of Medicine and Health Sciences, NTNU Norwegian University of Science and Technology, Trondheim, Norway; 4 National Research Center for the Working Environment, Copenhagen, Denmark; 5 Department of Public and Occupational Health, Amsterdam UMC, Vrije Universiteit, Amsterdam, Netherlands; 6 Unit kindergarten, Moss Municipality, Moss, Norway; 7 Kanvas Foundation, Oslo, Norway

**Keywords:** child day care centers, mental health, musculoskeletal health, occupational health, randomized controlled trial, workplace intervention, work-related fatigue

## Abstract

**Introduction:**

In Norway, the early education and care (ECEC) sector has the highest rate of work-related fatigue and sickness absence across all sectors. Work in ECEC can be characterized as physically and emotionally demanding, which may lead to fatigue and the two most common causes of sickness absence in the ECEC sector, i.e., musculoskeletal and mental health issues. Improvements to the work environment can lead to better health for ECEC employees and improve ECEC quality. Therefore, work environment, employee health, and ECEC quality go Hand-in-Hand. This paper presents the protocol for conducting a trial to evaluate the effectiveness and implementation of the tailored participatory Hand-in-Hand workplace intervention.

**Methods and analysis:**

The study is a 1-year pragmatic cluster-randomized controlled trial with a wait-listed control group. Nineteen ECEC centers were recruited from two Norwegian municipalities, with an estimated 350 employees. The intervention is facilitated by external consultants and includes one workshop and two workplace visits. This includes training the employees in the Hand-in-Hand toolbox, a systematic method for developing workplace measures. Data collection will consist of accelerometer and heart rate measurements, questionnaires, diaries, interviews and registry data; baseline measurements were conducted in October/November 2025. The primary outcome of the effect evaluation will be work-related fatigue. The effectiveness of the intervention will be analyzed using multilevel analyses. In addition, a process evaluation will assess intervention implementation to help understand why the intervention is effective (or not), and for whom and under which circumstances.

**Discussion:**

The intervention adopts an interdisciplinary approach, integrating emotion work, sensor-based measurements of physical workload, and quality in early childhood education and care. The study aims to provide theoretical, methodological, and practical insights into how to design and implement a complex intervention in an ECEC setting.

**Trial registration:**

https://www.isrctn.com/ISRCTN81684777, identifier: ISRCTN81684777.

## 1 Introduction

In Norway, early education and care (ECEC) is an integral component of the educational system, with a strong emphasis on enhancing its quality due to its lasting impact on school achievement and overall adult outcomes, such as higher education attainment and income (1, 2). The quality in ECEC is influenced by several factors including the caregiver-child ratio, personnel stability, and the capability of staff to create a caring environment (3–6). However, ECEC staff face significant health concerns affecting the quality of care; they report the highest rates of work-related fatigue and sickness absence among all sectors, alongside frequent mental health issues and musculoskeletal complaints (7, 8). These challenges do not only affect the quality of care, they also impact the employees' quality of life, and lead to increased costs for employers and society. Hence, addressing these health issues is essential not only for the wellbeing of ECEC staff but also for the sustainability and effectiveness of the early education system in Norway.

The health issues faced by ECEC staff are likely related to their work. ECEC workers report high physical workloads—including lifting in awkward postures, kneeling and forward bending, while lacking possible health promoting high-intensity physical activity (7, 9, 10). The work is also emotion work, i.e., requiring emotion regulation and the display of desired emotions in interaction with children, colleagues and parents, which can be perceived as demanding (11, 12). These work demands are associated with increased fatigue, higher risks of mental and musculoskeletal health issues, and long-term sickness absence (11–18). We theorize that sustained fatigue mediates the effects of physical workload and emotional demands on the risk of musculoskeletal and mental issues—the leading causes of sickness absence in the ECEC sector (19–21).

Despite these demands, ECEC work also holds health-promoting potential. Introducing high-intensity physical activity at work has been shown to reduce fatigue, improve recovery, and has potential to mitigate downstream health effects (20, 22). Moreover, emotion work can have positive effects on health. Mastering emotion work by fulfilling one's professional role and altering one's inner feelings to match desired emotions (experiencing a low degree of emotional dissonance), along with frequent display of positive emotions can increase job satisfaction, sense of accomplishment and lead to a confirmation of identity, thus fostering wellbeing (23). Similarly to the positive impact of high-intensity physical activity, we theorize that mastering emotion work will increase energy levels, thereby mitigating downstream health effects.

Taken together, a dual approach that reduces health-damaging aspects of physical workload and emotion work, while harnessing their health-promoting aspects, could enhance employees' health. To our knowledge, only two previous studies in ECEC have targeted physical workload, each supporting one aspect of this dual approach (22, 24). The first is the TOY–project which reduced health-damaging physical workload, i.e., lifting, carrying, and forward bending for the employees by encouraging the children to be active participants in daily tasks (e.g., climbing up on the changing table or dressing themselves), which lead to lower musculoskeletal-related sickness absence (24). The second is the Goldilocks-childcare study that aimed to promote health by creating a “just right” physical workload, by increasing time in high intensity occupational physical activity (≥60% heart rate reserve) through playing games with the children (22). The study led to increased energy levels and a reduced need for recovery among employees (22). The promising results of both studies resulted in the TOY-project being scaled up to be delivered to all 350 ECEC centers in Copenhagen, now including an element of high-intensity physical activity (25). This dual approach seems promising for improving ECEC employees' health, and aligns with the Goldilocks Work paradigm, i.e., to (re)design work to not only focus on reducing exposures, but to create a “just right” combination of work exposures for improved health.

Not only did the TOY-and Goldilocks-childcare studies show potential to improve physical workload, the projects proposed measures that, both directly and indirectly, can have a positive impact on ECEC quality. ECEC quality includes several aspects: process quality [e.g., staff-child interactions (26, 27)], outcome quality [e.g., children's outcomes regarding motor, social, cognitive and emotional development (28)] and structural quality [e.g., physical environment, group organization, staff competence (26, 29)]. The TOY-project directly increased quality by involving children in daily tasks, thus increasing the quality of staff-child interactions (process quality) and supporting children's independence and motor skills (outcome quality) (24, 30). The Goldilocks-childcare study approached the same goals through active game-playing with the children (22). Additionally, ECEC quality can indirectly improve through improved employee health and more stable staffing via reduced sickness absence and turn-over (structural quality), which can facilitate creating a caring environment characterized by high-quality staff-child interactions (4, 5). This highlights that the work environment, employee health, and ECEC quality are interdependent and mutually reinforcing, and thus go Hand-in-Hand.

Beyond improving physical workload, there is a call for interventions that address emotion work (31). Building on the premise that the work environment, employee health and ECEC quality are interdependent, we extend this application to emotion work. Inspired by the Goldilocks Work paradigm—and recognizing that emotion work has both demanding and rewarding facets—we propose a dual approach: reduce its demanding aspects while enhancing its rewarding aspects. This “just right” exposure of emotion work may promote health as well as improve ECEC quality. To our knowledge there are no intervention studies among ECEC workers that set out to create measures focusing specifically on health promotion through a “just right” exposure to emotion work that simultaneously considers ECEC quality.

Building on findings from the TOY-and Goldilocks-childcare studies, there is a need for interventions that address physical workload and emotion work in ways that are feasible and meaningful within everyday ECEC practice. Participatory approaches, where employees and leaders collaboratively identify challenges and develop locally adapted solutions, may increase implementation success and sustainability of work environment changes (32, 33). Inspired by the Goldilocks Work paradigm, we developed the Hand-in-Hand intervention through stakeholder involvement and a feasibility study following a framework for development of complex interventions (34). The purpose of the Hand-in-Hand intervention is to support ECEC centers in organizing work to improve health and ECEC quality through a “just right” combination of physical workload and/or emotion work exposures.

This paper will describe the protocol of a cluster randomized controlled trial aiming to evaluate the effectiveness and implementation of the Hand-in-Hand intervention on employee health, work environment factors and indicators of ECEC quality.

## 2 Methods

Reporting of this protocol paper was guided by the SPIRIT guidelines (35).

### 2.1 Trial design

The study uses a pragmatic cluster-randomized controlled trial design with a wait-listed control group (Figure 1). The study is an organizational intervention, in which the ECEC centers form the clusters. The intervention will last for 1 year. Measurements will take place at baseline (T0), at 3 months (T3), at 6 months (T6), at 9 months (T9) and at 12 months (T12). Data gathering commenced in October 2025.

**Figure 1:**
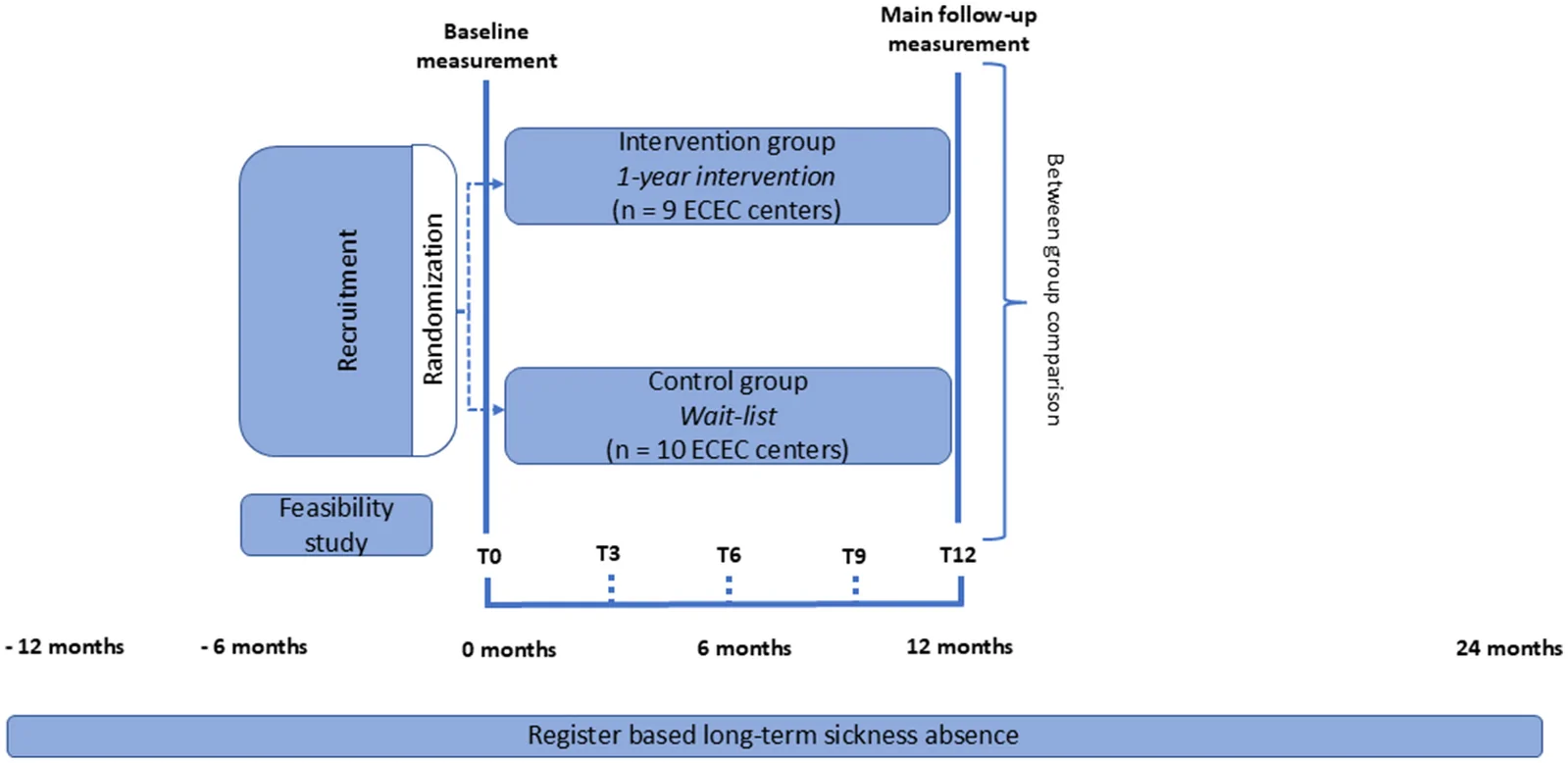
Study design and timeline: a pragmatic cluster-randomized controlled trial with a wait-listed control group. Comprehensive assessments of work environment factors, health outcomes, and quality in ECEC will be conducted at baseline (T0) and after the intervention year (T12), with less-comprehensive assessments performed at T3 to T9. Throughout the intervention period, we will collect data for the process evaluation. After the intervention year, the controls may receive the intervention. Physician certified sickness absence (>2 weeks) will be obtained from national registers.

### 2.2 User and public involvement

Stakeholder collaboration is integrated throughout the development and implementation of the intervention. Early dialogue with municipality and ECEC leaders shaped the Hand-in-Hand intervention by including emotion work alongside physical workload as a main theme. Stakeholders and employees from a private ECEC company provided feedback on the intervention and data collection methods during the feasibility study. To guide the implementation, we established a multi-stakeholder working group that includes ECEC owners, Human Resources staff, and Health, Safety and Environment representatives and a community physician from the participating municipalities. The purpose of this group is to co-design rollout strategies, ensure feasibility and sustainability (e.g., integrating activities into regular working hours), and to act as a support function for the ECEC centers as well as a bidirectional communication channel for the research team.

### 2.3 Setting and participants

Four Norwegian municipalities were approached by the research team prior to finalizing the intervention for participation in the study. Of those four, Moss and Vestby municipalities agreed to participate. Moss and Vestby are adjacent municipalities who vary in population size and number of ECEC centers. Each ECEC center has a leader who sits in the center's Joint Consultation Committee, and who reports to the municipal ECEC leader, reflecting how Norwegian ECEC centers commonly are organized. The ECEC centers range in size from 43 to 124 children; all include children between the ages of 1 to 5 years. The ECEC centers are divided into departments or zones, based on the age of the children. The employees commonly work with one group of children.

To anchor the intervention within the whole organization, guided by the Individual, Group, Leader, Organization, Overarching context (IGLOO) framework (36, 37), information and recruitment meetings were held at various levels of the organizations in both municipalities. This included meetings with the municipal ECEC leaders, as well as with the ECEC center leaders and Joint Consultation Committees, and with the employees. All seven public ECEC centers in Vestby, as well as all 11 public ECEC centers and one private center in Moss, agreed to participate. Employees were then invited to participate in the individual data collection, including questionnaires, sensor-based physical workload measurements and interviews (described under the section Data collection and management).

All employees working with children, and ECEC center leaders, were eligible to answer the baseline questionnaire. Employees who worked ≥40% of a full-time position and intended to work in the ECEC center at least through February 2026 were included in the sensor-based measurements. Pregnant employees with pregnancy-related adjustments to their work tasks, and employees with known allergies to adhesive tape, were excluded from sensor-based measurements.

### 2.4 The Hand-in-Hand intervention

#### 2.4.1 Intervention development

The intervention builds on the TOY-project in which changes in the work environment are guided by improvements in quality of care (24, 30). In addition, the intervention is inspired by the Goldilocks Work paradigm where key aspects include (1) (re)designing jobs to have a “just right” combination of physical and psychosocial occupational exposures, rather than aiming for zero exposures, (2) organizing work so that it leads workers into “just right” behaviors, rather than relying mainly on individual motivation and resources, (3) to explicitly have a positive impact on health, rather than mainly focusing on reducing risk and removing negative impacts (38) .

The intervention was developed based on the framework for development of complex interventions by Skivington et al. (34). Central are the consideration of the intervention context and the development of the intervention based on previous research evidence. In the ECEC sector, previous interventions have induced change by using 1) a participatory approach, 2) integrating measures into work tasks, and 3) performing a needs mapping (22, 24, 39). These three components, which are also highlighted in the Goldilocks work paradigm (38), are used in the Hand-in-Hand intervention. In addition, the intervention is guided by the COM-B (Capability, opportunity, and motivation-behavior) model to generate behavioral change (40). Project stakeholders were involved in the intervention development as recommended by the framework (34).

As suggested by the framework, a program theory has been developed which was refined after the feasibility study (Figure 2) (34). This model distinguishes the components of the COM-B model (40) and presents how the intervention may contribute toward improvements in the work environment, improved employee health, and improved ECEC quality. The program theory helps to guide the effect and the process evaluations of the intervention.

**Figure 2:**
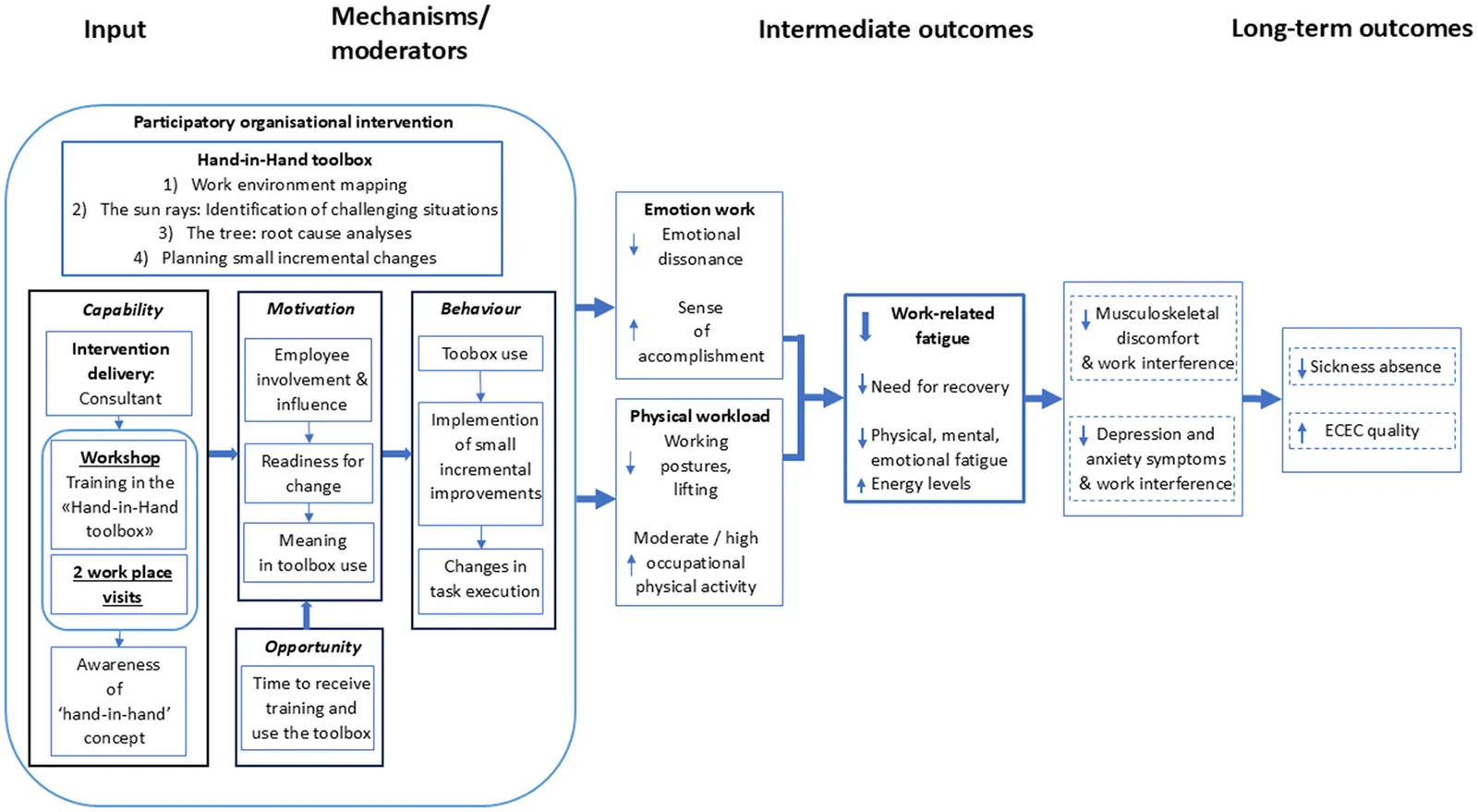
The program theory presents the intervention activities, possible mechanisms and moderators and hypothesized intermediate and long-term outcomes.

#### 2.4.2 Feasibility study

In line with recommendations, a feasibility study was conducted prior to the trial (34). The aim was to test and further develop the intervention content and data collection methods. Employees from three ECEC centers in Oslo from a private ECEC organization participated in baseline measurements, a workshop and one workplace visit. The ECEC centers had a total of 62 employees where 42 completed a baseline questionnaire and 48 completed sensor-based measurements. Intervention activities were facilitated by three ECEC advisors working for the organization. Based on feedback from the ECEC advisors, as well as ECEC center leaders and employees, the feasibility study led to adjustments to the intervention content and the structure of implementation activities to further fit the ECEC context. This included adjustments to the workshop format, the conceptualization of the Hand-in-Hand toolbox, the design of the templates used in the toolbox to systematically create measures, receipt of the needs mapping at the workshop, and the broad and tailored format of the workplace visits.

#### 2.4.3 The Hand-in-Hand toolbox

The core of the intervention is the Hand-in-Hand toolbox that covers the two themes physical workload and emotion work. The toolbox consists of a needs mapping (a group report of the baseline measurements) and three templates: (1) “The sun rays” to identify work situations in need of improvement, (2) “The tree” to conduct a root cause analysis of these work situations and to agree on how to address the root cause, and (3) “Planning of small incremental improvements” to build concrete solutions (41). The toolbox is intended as a continuously available resource throughout the intervention year, supporting ECEC centers in developing new measures aligned with one or both toolbox themes. The toolbox templates will not be made publicly available until after the intervention period to reduce the risk of contamination.

### 2.5 Intervention implementation

The intervention is implemented through one workshop and two workplace visits, held in each ECEC center. All intervention activities take place during working hours. Additionally, all ECEC centers are informed about the online resource “Intelligent physical exercise training at work” (42) and provided with the Goldilocks-games instructions. These games serve as an inspiration for activities with the children where the employees can increase time in high intensity physical activity (22).

The workshops and the workplace visits are held by consultants working at the Norwegian Labor and Welfare Administration (NAV) work life center, a public resource center that specializes in assisting organizations struggling with high sickness absence. The NAV consultants are trained by the principal investigator in the delivery of the workshop through a 4-h training session. During the training, the toolbox content and workshop educational material are presented and discussed along with supplementary materials to educate the consultants on the background of the intervention.

To make sure that resources in the IGLOO model are utilized (36, 37), the project's working group attended the workshop training session where their roles were presented (organizational level). The roles of ECEC center leaders (leader level), the centers' Joint Consultation Committees (group level) and each position (individual level) within the ECEC centers were presented to the ECEC center employees during the workshop.

#### 2.5.1 Workshop

The workshop was conducted during working hours for each ECEC center and all their employees in November 2025. The presentation and toolbox-templates used for the workshop were provided by the researchers. The content of the workshop is described in Figure 3. The aim of the almost 4-h lasting workshop was to train the employees in the Hand-in-Hand toolbox and to raise awareness on how the work environment, employee health, and ECEC quality go Hand-in-Hand. Each ECEC center focused on either physical workload or emotion work during the workshop, chosen through a vote.

**Figure 3:**
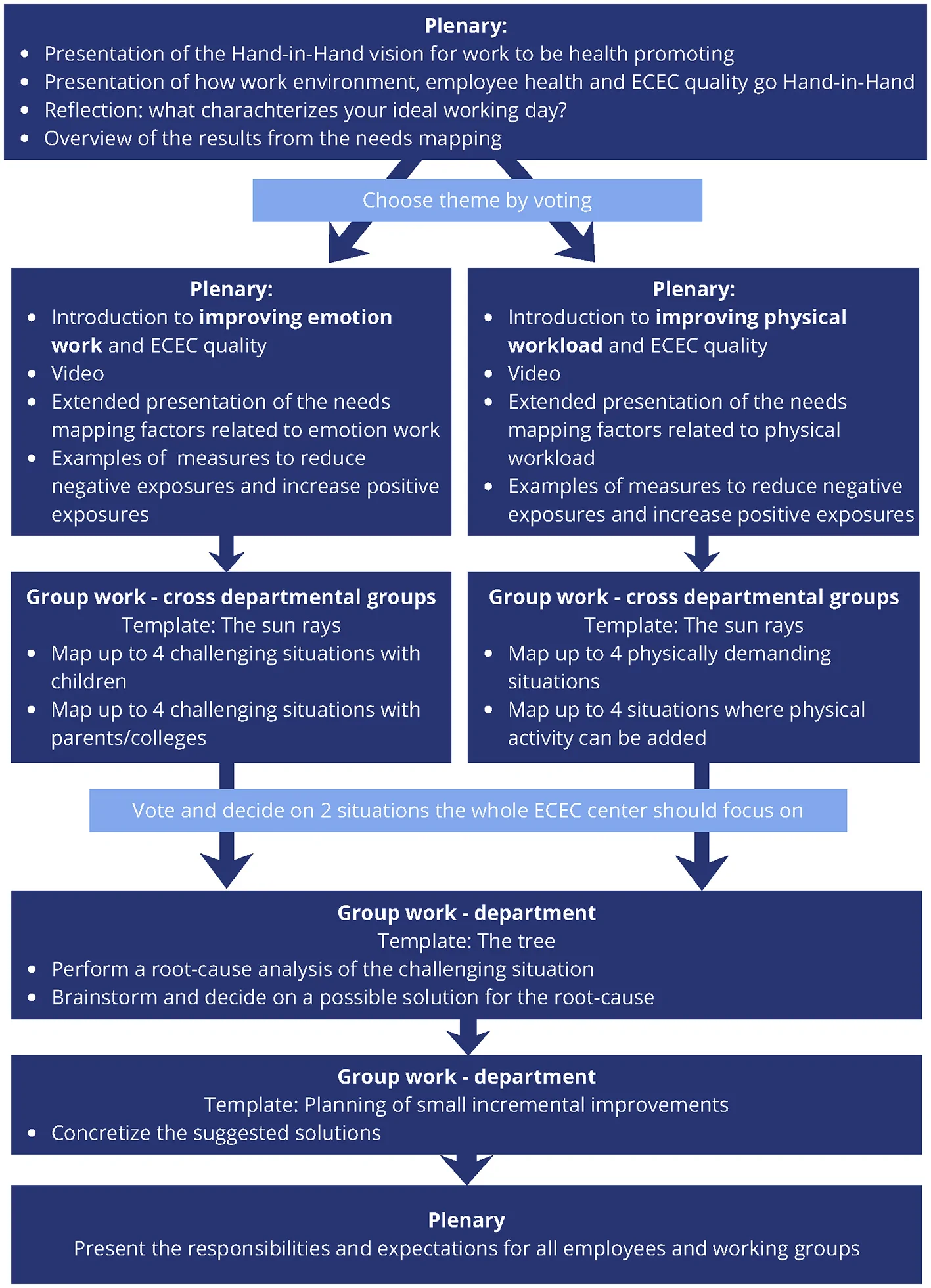
An overview of the workshop activities.

#### 2.5.2 Workplace visit

The aim of the two workplace visits is to evaluate whether the ECEC centers have started using the Hand-in-Hand toolbox, identify needs for improvement, and make a follow-up plan. The specific plan for the workplace visit is agreed upon by the consultant and the ECEC center tailored to their needs. For example, the visit can include, but is not limited to observation, a meeting with the center leader and department leaders, and a meeting with other staff members. A guideline of what the workplace visit should entail, and what potential observations and meetings should cover, is provided to the consultants.

### 2.6 Control group

The ECEC centers in the control group will receive a group report based on the baseline and 1-year follow-up measurements. This is given both to minimize the risk that potential between-group differences may be a result of research participation effects in the intervention group (43), as well as for recruitment motivation. ECEC centers in the control group continue their usual practice and will be able to receive the intervention at their own request after the completion of the follow-up measurements.

### 2.7 Sample size calculation

To conduct a pragmatic cluster-randomized controlled trial we will require 246 participants (123 in each group), taking into consideration a drop-out rate of 15%. Calculations used data from the TOY-project conducted in ECEC-centers (24), where baseline mean Need for Recovery of 63 (SD ± 25; 0–100). We targeted an 11-point reduction to fall under the 54-point cut-off for increased mental-health risk (44), using *p* < 0.05, power = 0.80, intraclass correlation coefficient = 0.005, and accounting for clustering with an expected average of 15 participants per ECEC center (45). The computation was done using GLIMMPSE (version 2, https://glimmpse.samplesizeshop.org). Moss and Vestby municipalities were recruited as they had 19 interested ECEC centers with an estimation of 350 employees. Based on previous interventions, as well as our feasibility study, where participation ranged between 42 and 86% (22, 24, 46) we expected a participation rate of approximately 70% (*n* = 246).

### 2.8 Randomization and blinding

To account for potential contextual differences between the municipalities and center sizes, the ECEC centers within each municipality were stratified by size, based on the number of children (small <50, medium 50–75, large ≥76). Accordingly, the ECEC centers were randomized to either the intervention or the control group (1:1) using Excel [Microsoft Excel, Microsoft Office Professional Plus 2013, RANDOMBETWEEN (1–50)] to generate a computer-based random sequence. In total, nine ECEC centers (three in Vestby, six in Moss) were assigned to the intervention group and 10 ECEC centers (four in Vestby, six in Moss) were assigned to the control group. The randomization was performed after ECEC center recruitment and the employee information meetings but prior to employee recruitment, due to scheduling needs. Potential selection bias arising from individual recruitment after ECEC center randomization, will be checked by assessing whether the number of participating employees per ECEC differed between the intervention-arm and control-arm, whether baseline levels of work-related fatigue differed between the intervention and control-arm, and whether there is attrition asymmetry depicted in a CONSORT-Cluster flow diagram. Participant blinding was not possible as this is a participatory intervention where employees actively seek to find and implement solutions to their work environmental challenges. Researchers could not be blinded due to logistical reasons regarding time and sensor availability and the relatively small team of two researchers administering the project. Group allocation will be recoded after data collection, and researchers will be blinded during data analysis.

### 2.9 Data collection and management

Baseline and 1-year follow-up measurements consist of questionnaires and sensor-based measurements, i.e., accelerometer and heart rate (HR) measurements (Table 1). In addition, a short questionnaire will be administered at T3, T6, and T9. Registry data on physician-certified sickness absence (>16 days) will be collected for 3 years, beginning 1 year prior to the intervention, and will be combined with self-certified sickness absence obtained directly from the participating ECEC centers. Data collection for the process evaluation will include interviews with employees, a questionnaire administered to employees during the workshop, a questionnaire administered to the ECEC center leaders, and a questionnaire administered to the consultants. All data will be downloaded and stored on a secure server.

**Table 1:** The timeline for assessment, including primary and secondary outcomes, guided by the SPIRIT guidelines (35), indicating method of data collection.

Timepoint	Trial period						
	Pre-trial	Baseline	Workshop	3 months	6 months	9 months	1-year follow up
Assessments: primary outcome							
Work related fatigue		Q		Q	Q	Q	Q
Secondary outcomes							
Physical workload		S + Q		Q	Q	Q	S + Q
Emotional dissonance		Q		Q	Q	Q	Q
Compassion satisfaction and emotional demands		Q					Q
Musculoskeletal pain		Q		Q	Q	Q	Q
Depression and anxiety symptoms		Q		Q	Q	Q	Q
Awareness of Hand-in-Hand		Q					Q
MSP interference		Q					Q
Depression and anxiety interference		Q					Q
Turnover intention		Q					Q
Sickness absence	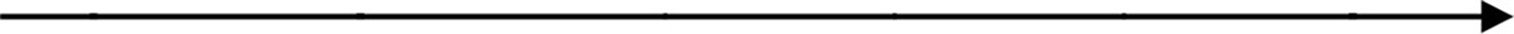						
ECEC quality		Q					Q
Process evaluation							
Consultant delivery			Q	Q 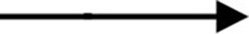	Q		
Work environment measures not related to Hand-in-Hand							Q
Readiness for change			Q				
Employee experience				I 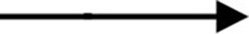	I		I
Toolbox use				Q	Q	Q	Q

S, sensor-based measurements; Q, Questionnaire; I, interview.

The intervention has been assessed as low risk, and to avoid overburdening participants we will not directly assess unintended adverse events. However, if such events occur, they will be captured through feedback sheets filled in by the NAV consultants after the workplace visits. Wearing the sensors to assess physical workload may cause some skin discomfort. To reduce any risk of skin discomfort during sensor attachment, participants are instructed to remove the sensors if they experience soreness under the sensors.

### 2.10 Outcomes

Differences between the intervention and control groups in changes from baseline to 1-year follow-up will be evaluated for the primary and secondary outcomes.

#### 2.10.1 Primary outcomes

The primary outcomes are measures of self-reported work-related fatigue, assessed by the Need for Recovery scale and four single-item questions on physical, emotional, and mental fatigue, and on energy levels. Need for Recovery is assessed with an 11-item scale with dichotomous answers (“0-No” / “1-Yes”) (47). The need for recovery score is the number of positive answers on the items, rescaled to 0–100, where higher scores reflect a higher need for recovery. The scale has been shown to have good reliability (rho = 0.86), sufficient content validity, and has predictive validity for sickness absence (47). The four single item fatigue questions have similar phrasing, e.g., “*How emotionally fatigued have you felt at work*?” and “*How much energy have you had at work*?” based on the single-item fatigue questions developed by van Hooff et al (48). Each of the questions is answered on an 11-point numeric rating scale from “0-Very little” to “10-Very much” (48). The single-item questions by van Hooff et al (48) have shown sufficient convergent and discriminant validity (48). Work-related fatigue is assessed at all five measurement time-points (Table 1).

#### 2.10.2 Secondary outcomes

The secondary outcomes relate to changes in outcomes, that according to the program theory, are more proximal or more distal to the intervention than work-related fatigue (Figure 2). The outcomes relate to awareness of how the work environment and ECEC quality go Hand-in-Hand, changes in physical workload and emotion work, and changes in health-related outcomes, i.e., depression and anxiety symptoms, musculoskeletal symptoms, interference of these symptoms with work performance, sickness absence, and changes in ECEC quality.

*Awareness of how work environment and ECEC quality go hand-in-hand* is mapped by three self-designed statements that participants consider in how far they agree with, on a 5-point scale from 1-Strongly disagree to 5-Strongly agree.

*Emotion work* is assessed by different self-reported dimensions. Moving toward “just right” emotion work entails a simultaneous increase in a sense of accomplishment and reductions in emotional demands and emotional dissonance. Emotional demands are assessed using a 3-item scale from COPSOQ with answers on 5-point scale from “1-Always” to “5-Never/hardly” or “1-To a very large extent” to “5-To a very small extent” (49). Emotional dissonance is assessed using four items from the Frankfurt Emotion Work Scales with answers on a 5-point scale from “1-Seldom or never” to “5-Several times an hour” (11, 50). A sense of accomplishment is captured using the ProQoL-Compassion Satisfaction subscale that consists of 10 items answered on 5-point scale from “1-Never” to “5-Very often” (51).

*Depression and anxiety symptoms* will be assessed by the Hopkins Symptom Checklist (HSCL-5) that consists of assessing the degree to which participants are bothered by symptoms on a 4-point scale from “1-Not at all” to “4-Extremely” (52). *Interference of these symptoms in work performance* are assessed by two items inspired by the Short Form Health survey (SF-36) (53).

*Musculoskeletal pain* are assessed on a numeric rating scale from “0-no pain” to “10-worst imaginable pain” for nine different bodily regions (neck, shoulder, elbows, hands/wrists, lower back, hips, knees, feet/ankles, and headache/migraine) based on the Nordic questionnaire (54). *Musculoskeletal pain interference in work performance* is assessed by a single item from the SF-36 for each body region about whether participants had to reduce activities at work due to the symptoms in that area. Responses are recorded as “0-No” or “1-Yes” (53).

*ECEC quality* will be assessed by adapting questions from the EBBA observational tool that has been developed for the Norwegian ECEC context (55). Topics that will be assessed are indoor and outdoor physical layout of the ECEC, indoor climate (structural quality), children's participation in the physical environment, staff-child interaction, play, parental cooperation, and situations (mealtimes, bathroom visits, changing clothes; process quality).

*Turnover intention* will be captured by two single item questions, one on thoughts on leaving the current position answered on a scale from “1-Never” to “4-Often” (56) and one on intentions to seek employment elsewhere with possible answers “1-No.” “2-Yes, I am looking now” and “3-I intend to look” (57).

*Physician-certified sickness absence* (>16 days) will be assessed by registry data from the NAV. This is trajectory data, that tracks sickness absence from day to day. *Self-certified sickness absence* will consist of the number of sickness absence days per total number of employees at the ECEC center per 3 months.

*Physical workload* will be measured by sensor-based recording of body postures, and intensity and type of occupational physical activity (see following section), as well as self-perceived physical exertion and number of lifts per day. Moving toward a “just right” physical workload will be evaluated by reductions in the body postures arm elevation, trunk forward bending, and kneeling, as well as self-perceive physical exertion and number of daily lifts, coupled with increases in time spent in moderate and high intensity physical activity. Working with elevated arms above 60 degrees, forward bending beyond 30 degrees, and kneeling will be assessed in duration and frequency. Intensity of physical activity will be determined by cardiovascular load [time spent in moderate and high intensity at respectively HR ≥40% and ≥60% of relative heart rate reserve (58, 59)] and type of activity will be categorized as time spent walking, running, stair climbing or cycling. In addition, employees self-reported number of daily lifts of an item or child weighing more than 7 kg (answers on a 5-point scale: “1-At least 20 times a day,” “2–5 to 19 times,” “3–1 to 4 times,” “4-none”). This is a question adapted from a questionnaire held by Statistics Norway (60). Lastly, self-perceived physical exertion will be assessed by the Borg scale CR-10 which is a 15-point scale with some anchors along the scale (61).

##### 2.10.2.1 Sensor-based assessments of physical workload

Participants will be asked to wear four accelerometers and one HR monitor for seven continuous days. To reduce the risk of measurement reactivity bias neither of the sensors provide immediate feedback (62).

*Body postures and type of occupational physical activity* are processed using the Motus system which entails the integration of four accelerometers (SENS motion; SENS innovation ApS, Copenhagen, Denmark) and the Motus smartphone application (Motus–Work Move Measure; SENS Innovation ApS, Copenhagen, Denmark) (63). The Motus system is both a usable and feasible method for accelerometer administration (64, 65). The SENS accelerometer records linear acceleration in three dimensions (an x, y and z axis) with a range of ± 4 G, it has a sampling frequency of 12.5 Hz and an internal storage capacity of 10 days. The sampling frequency of 12.5 Hz has previously been compared to a sampling frequency of 25 Hz and although the lower sampling frequency might lead to an underestimation of running, the overall agreement is good (63).

The accelerometers will be mounted by the research team on the right thigh (midway on the line between the base of the patella and anterior inferior iliac spine), the trunk (posteriorly, at the level of the process spinosus of T4 and T5), the dominant arm (laterally, just distal to the deltoid insertion) and on the right calf (on the most inferior part between the medial and lateral head of gastrocnemius). If participants are absent on the day of mounting, they can self-attach the sensors guided by the Motus app. After attachment, the sensors are calibrated through the app. The participants are asked to log into the app daily to synchronize the sensor data to the cloud via Bluetooth. To categorize data into work, leisure and sleep, participants are asked to register day type (i.e., workday, non-workday) along with work and sleep time.

A software tailored to the use of SENS motion accelerometers based on Acti4 (63) will categorize movement behaviors based on movement intensity and orientation. Movement outcomes used in this evaluation have previously been validated in both laboratory settings and during free-living for walking, lying, sitting, cycling, stairs, moving, standing, and for the body postures kneeling, arm elevation and forward bending of the trunk (66–69).

*Cardiovascular load* is assessed by HR measured using the single-lead ECG monitor Fibion flash (Fibion flash; Fibion Inc, Jyväskylä, Finland) connected to an electrode adaptor (Movesense Ltd, Vantaa, Finland) attached to two electrodes (AMBU BlueSensor VLC; Ambu, Denmark). The Fibion flash measures beat to beat RR interval data at a 1 ms resolution. Kaasa Data collector (Data collector; Kaasa solution Gmbh, Düsseldorf, Germany) is used to administer the sensor recording and data downloading trough Bluetooth. Throughout the measurement period the data is stored locally on the sensor. The Fibion flash is mounted diagonally on the left side of the chest in the space between costae 1 and 4, with the highest electrode just below the acromial end of the clavicle. If necessary, participants are asked to shave before attaching the electrodes. Due to the 72-h durability of the Ambu electrodes, the participants are asked to change electrodes every other day. The Fibion flash is a resale of the Movesense flash sensor (Movesense Flash; Movesense Ltd, Vantaa, Finland). Although we are not aware of any validation studies for either sensor, there are studies comparing similar Movesense products (the Movesense HR+ and Movesense medical) placed in a chest strap to a 3-lead or a 12-lead ECG. The studies concluded that the products are in good agreement with their reference measurement for HR and RR-intervals (70–72).

#### 2.10.3 Background information

Demographic and health-related information are assessed at baseline by questionnaire: gender, body mass index (BMI-calculated from weight/height in meters squared), age (in years), civil status, educational level, and smoking status (yes/no). Work-related information includes work experience (in years) and occupational title.

### 2.11 Process evaluation

The process evaluation will be guided by the model developed by Nielsen and Randall (73) as well as the COM-B model for behavioral change (40). The evaluation will be conducted using mixed methods. To evaluate fidelity, we will investigate if the intervention was delivered in accordance with the protocol, and how the intervention was received by the participants. Delivery includes dose delivered, quality and content as defined by Ferm et al. (74). Receipt includes responsiveness as defined by Ferm et al. (74) and dose received and reach as defined by Linnan and Steckler (75). Data collection (Table 1) consists of questionnaires and feedback sheets from consultants regarding the workshop, workplace visits, and their own evaluation of the implementation of these. Further quantitative evaluation will consist of a readiness for change questionnaire (76), answered as a part of the workshop, and questions regarding the use of the toolbox in the follow-up questionnaires every 3 months for the intervention group. In addition, a questionnaire will be sent to the ECEC center leaders at the end of the intervention period to map any work environment-related measures they have taken (that were not related to the Hand-in-Hand project) during the intervention period. For the qualitative evaluation we will conduct two semi-structured interviews with two employees from each intervention ECEC center. The employees will be selected by the ECEC center leaders based on availability and can vary between interviews. Inclusion criteria for the interviews are participation in the baseline measurements and the workshop. To reduce the risk of recall bias, the first interview will be shortly after the first workplace visit to gain insight into the experience of the workshop and workplace visit. The second interview will be held after the 1-year follow-up to discuss the overall experience of the intervention.

### 2.12 Data analysis

Between-group differences will be analyzed using multilevel analyses, i.e., generalized estimating equations (GEE), that consider repeated measures within individuals that are nested in different ECEC centers (77). GEE analyses are also well suited to handle missing data. Each model, for both the primary and secondary outcomes, will test the group (intervention/control) *x* time interaction, and will be adjusted for randomization stratification factors, i.e., municipality and ECEC center size, and baseline values of the outcome. Analyses will be conducted according to intention-to-treat. The analyses for all the primary and secondary outcomes will include all participants, except for outcomes assessed with sensors, which will involve fewer participants due to exclusion criteria. If data are not missing completely at random, informative (non-ignorable) dropout will be accounted for in the analysis (78).

Sensitivity analyses will be performed in which we stratify ECEC centers by theme they have worked with, i.e., emotion work only, physical workload only, and both themes. Additionally, we will conduct a per-protocol analysis, including only those ECEC centers who complied with the study protocol (i.e., having received the workshop, having received two workplace visits, and where the majority of the participants report having used the toolbox throughout the intervention year).

Data obtained for the process evaluation will be analyzed using comparative analyses according to the degree of implementation success in the ECEC centers and/or their ability to create change in the primary and/or secondary outcomes. The quantitative data in the process evaluation will be compared descriptively, and the qualitative data will be analyzed using reflective thematic analysis.

## 3 Discussion

This study protocol describes the methods to evaluate the effectiveness and implementation of the tailored participatory workplace intervention: Hand-in-Hand. The purpose of the Hand-in-Hand intervention is to support ECEC centers in organizing work to improve health and ECEC quality trough a “just right” combination of physical workload and/or emotion work exposures.

### 3.1 Strengths and limitations

A clear strength of this intervention is the tailored participatory approach where a needs mapping is used to develop measures focusing on adjusting current work tasks. The study uses an interdisciplinary approach where physical workload and emotion work are seen in relation to ECEC quality, making the actions valuable to implement for the employees and ECEC center leaders, as well as stakeholders in the municipality. It is also of scientific value as it will deepen our understanding of the interplay between work environment factors and quality in ECEC. The in-depth involvement of feasibility study contributors and stakeholders is another strength, as it has helped develop an intervention that fits the ECEC context.

Physical workload is commonly reported using questionnaires, which can lead to an inaccurate description of exposures as such methods are prone to recall bias and depend on a subjective evaluation of movement patterns and duration (79, 80). By assessing body postures and physical activity type and intensity through sensor-based measurements, our study will provide more accurate knowledge of physical workload in the ECEC sector. Obtaining register-based data on sickness absence is another methodologically strong outcome measure planned for this trial. The 1-year follow-up period is considered a strength as it can take time for workplace changes, and the effect of changes to occur. The 1-year follow-up will also reflect the sustainability of the Hand-in-Hand intervention.

The thorough process evaluation planned for this intervention is a strength as it will provide insight into which aspects of the intervention were successful and why, and factors that may facilitate or hinder future implementation. This knowledge can be used for both further potential implementation of the Hand-in-Hand intervention, and for workplace implementation research in general.

The results of the Hand-in-Hand intervention will be generalizable to other ECEC staff and centers in Norway. The use of external consultants from NAV work life center to conduct the workshops and workplace visits, and the relatively short duration of these activities, increases both generalizability and sustainability as it permits guidance during working hours from a free and state funded agency. The knowledge obtained from the project may also be generalizable to other professions where both physical workload and emotion work is experienced as demanding and the workforce is female dominated.

There are some limitations to this study. Due to scheduling needs, randomization was done prior to the consent of individual participants for data collection, and the participants were not blinded to which group they were allocated to. This may lead to a selection bias and a lower participation rate of participants in the control group. Another limitation is the inability to blind researchers to group allocation during data collection. Although we measure body postures and physical activity type and intensity with sensor technology, we have no means to measure lifting and manual handling by sensors, and they are instead measured by questionnaire. Lastly, using self-reports rather than observation to assess ECEC quality, may lead to biased estimates due to self-assessment bias.

### 3.2 Impact

The potential implications of a successful intervention can be far reaching, and experienced at an individual level (employee health), a group level (work environment and quality in ECEC centers) and at a societal level. If the Hand-in-Hand intervention shows effectiveness, it will be important for policy makers aiming to reduce sickness absence and improve quality in ECEC. The intervention can then be rolled out in more ECEC centers in other municipalities and contribute to reducing the economic cost of sickness absence for ECEC owners (i.e., municipalities and private ECEC companies) and the large societal costs related to sickness absence and healthcare. Additionally, incorporating the project's findings in occupational health service and teacher training will improve ECEC quality in the future. High-quality ECEC at pre-school age can improve child development which, in turn, has been shown to have lasting positive impacts for school achievement and into adulthood (1, 2). Furthermore, improving health and working conditions among ECEC workers, and reducing sickness absence, will help secure adequate staffing for ECECs in the future and reduce withdrawal from the workforce.

### 3.3 Ethics and dissemination

The study will be performed in accordance with the Declaration of Helsinki. All participants will provide written informed consent, and data handling will be according to the General Data Protection Regulation (GDPR). The project has been approved by the Regional Committee for Medical and Health Research Ethics in Norway (Committee South-East C, ref number: 878385) and has been reported in the Norwegian Agency for Shared Services in Education and Research (SIKT).

We will organize a national seminar at the end of the project to present the main project results; lessons learned and provide recommendations for future initiatives. Scientific dissemination of the results from the study will be published in peer-reviewed Open Access journals and presented at national and international conferences.

### 3.4 Protocol amendments after trial commencement

After trial commencement, yet before data analysis, we implemented minor protocol refinements and updated the trial registry accordingly for transparency. Specifically, we specified one primary outcome, i.e., work-related fatigue assessed by single items and Need for Recovery, with all other pre-listed outcomes designated as secondary outcomes; no study outcomes were added or removed. We also corrected the planned sample size from 210 to 246 participants, to account for anticipated attrition and preserve statistical power. Concurrently, we addressed under-reporting at initial registration by adding four outcomes that had been included in the ethics-approved protocol, specified outcome assessment schedules in line with SPIRIT guidelines, and clarified (without changing) the eligibility criterion that only pregnant workers with adjusted tasks are excluded from the ergonomic evaluation. All refinements and clarifications were within the scope of prior ethics approval and did not require additional review.
